# Serogroup Y Meningococcal Disease, Colombia

**DOI:** 10.3201/eid1406.071357

**Published:** 2008-06

**Authors:** Clara Inés Agudelo, Olga Marina Sanabria, María Victoria Ovalle

**Affiliations:** * Instituto Nacional de Salud, Bogotá, Colombia

**Keywords:** Meningococcal infection, meningitis, serogroup Y, letter

**To the Editor:** *Neisseria meningitidis* is the etiologic agent of outbreaks, epidemics, and sporadic cases of meningitis or meningococcemia. Such infections have high illness and death rates, especially in children <5 years of age and adolescents. *N. meningitidis* serogroups A, B, C, Y, and W135 cause most meningococcal disease worldwide (*1*).

In Colombia, public health notification is required for all cases of invasive meningococcal disease. This reporting system is supported by a laboratory-based surveillance network for acute bacterial meningitis that has been coordinated by the Microbiology Group at the Instituto Nacional de Salud since 1994 (*2*,*3*). Clinical laboratories in Colombia submit isolates with associated information including geographic origin, specimen source, age, sex, and clinical diagnosis of the patient. Identification is confirmed by traditional phenotypic methods (*4*). Isolates are serogrouped by agglutination using commercial antisera (Difco, Detroit, MI, USA, and Becton Dickinson, Franklin Lakes, NJ, USA) and subtyped by dot blot with monoclonal antibodies (RIVM, Bilthoven, the Netherlands, and Institute Adolfo Lutz [IAL] São Paulo, Brazil) (*5*). Antimicrobial drug susceptibility testing for penicillin and rifampin is performed by the agar dilution, according to Clinical and Laboratory Standards Institute methods (*6*); for the breakpoints, we used those recommended by the Mesa Española de Normalización de la Sensibilidad y Resistencia a los Antimicrobianos (MENSURA) group (*7*). The reference laboratory participates in an external quality assurance program coordinated by the Pan American Health Organization (Sistema Regional de Vacunas [SIREVA] II, PAHO, Washington, DC, USA) with the Carlos III Institute, Madrid, Spain, and the IAL.

From 1994 through 2006, 434 *N. meningitidis* isolates were received by the Microbiology Group, from 22 of 35 departments (political divisions) and the Capital District: 119 (27.4%) from Antioquia, 117 (27.0%) from Bogotá, DC, 72 (16.6%) from Valle, 25 (5.8%) from Risaralda, 21 (4.8%) from Caldas, and 80 (18.4%) from 18 other departments. Distribution by department is published at the Institute’s website (www.ins.gov.co) (*8*). According to public health reports, the reference laboratory is receiving ≈27% of the clinical case isolates. A slight majority (53.8%) were cultured from male patients. The age of patients was available for 396 isolates: 254 (64.1%) were <1–9 years of age, 71 (17.9%) 10–19 years, 41 (10.4%) 20–39 years, 21 (5.3%) 40–59 years, and 9 (2.3%) >59 years. Three hundred ninety-two isolates (90.3%) were recovered from cerebrospinal fluid and 42 (9.7%) from blood cultures. The diagnosis for 420 (96.8%) patients was meningitis; 11 (2.5%) patients had sepsis or bacteremia, and 3 (0.7%) had other invasive diseases (pneumonia, encephalopathy, or cellulitis).

Serogroup distribution was 338 (77.9%) group B, 42 (9.7%) group C, 40 (9.2%) group Y, and 2 (0.5%) group W135; 12 isolates were nongroupable. There was little annual variation for groups B and C, but there was an unexpected increase in serogroup Y (Figure), from 0% in 1994 to 50% in 2006. When the period 1994–2002 was compared with 2003–2006, this change was significant, increasing from 2.2% to 29.5% (p<0.001).

**Figure:**
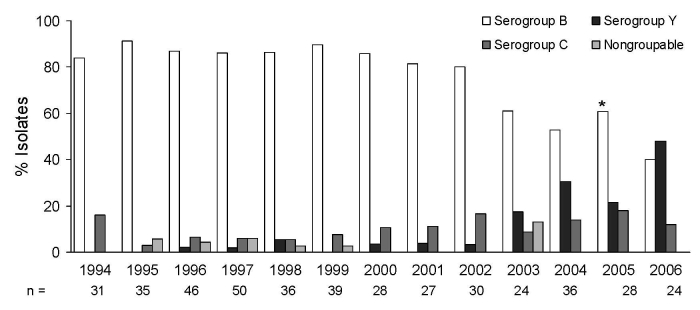
*Neisseria meningitidis,* serogroup distribution by surveillance year, N = 434. *p<0.001.

Antimicrobial drug–susceptibility testing showed that 17% of the isolates had intermediate resistance to penicillin (MIC 0.125–1.0 μg/mL) and 0.5% high resistance (>2.0 μg/mL); only 1 isolate was resistant to rifampin (>4.0 μg/mL). Penicillin resistance was not associated with any specific serogroup.

From the 40 serogroup Y isolates, 22 (55%) were from Bogotá, DC, 5 (12.5%) from Antioquia, 5 (12.5%) from Valle, and the remaining 8 (20%) from 5 other departments. Age distribution of patients who provided the isolates was as follows: 15 (37.5%) were <1–9 years of age, 7 (17.5%) 10–19 years, 11 (27.5%) 20–39 years, 4 (10%) 40–59 years, and 3 (7.5%) >59 years.

Subtyping of the 40 Y isolates showed that 30 (75.0%) were serotype 14 with 3 different subtypes: 23 were Y:14:NST; 6 were Y:14:P1.5,2; and 1 was, Y:14:P1.10. Four (9.8%) were nontypeable (NT) with 2 subtypes: 3 were NT:P1.5,2, and 1 was NT:NST; the remaining 6 (14.6%) belonged to 5 other serotypes. Intermediate resistance to penicillin was found in 5% of the serogroup Y isolates, and one was resistant to rifampin.

From 1994 through 2005, the laboratory-based surveillance program identified serogroup B as the most frequently isolated serogroup that caused acute bacterial meningitis in Colombia. In 2003, there was an unexpected increase in serogroup Y (Figure), and by 2006 it was the most common serogroup in Colombia. Seventy-five percent of the isolates collected during 2002–2006 were recovered from male patients younger than 14 years.

An increase in serogroup Y has also been reported in Chicago, Illinois, where one third of meningococcal disease cases are caused by this serogroup (*9*). A similar increase has been reported in Canada (*10*). Both the United States and Canada have investigated genetic similarity, and circulating clonal types have been determined (*9*,*10*). Similar molecular studies with Colombian isolates are under way in collaboration with the Carlos III Institute from Spain under PAHO coordination. Our data demonstrate the importance of laboratory-based surveillance programs supported by active participation of clinical and public health laboratories.
